# The EPICOVID19-BS study: a web-based epidemiological survey in bariatric patients

**DOI:** 10.1007/s40618-024-02407-1

**Published:** 2024-06-10

**Authors:** F. Prinelli, F. Adorni, A. Giovanelli, M. Ravelli, S. Ceresoli, C. Asteria

**Affiliations:** 1 Epidemiology and Public Health Unit, Institute of Biomedical Technologies, National Research Council, Via Fratelli Cervi 93, 20054 Segrate, MI Italy; 2 National Institute of Obesity Cure (INCO)-Bariatric Unit, IRCCS, Policlinico San Donato, Piazza Edmondo Malan, 2, San Donato Milanese, 20097 Milan, Italy

**Keywords:** SARS-CoV-2, COVID-19, Severe obesity, Observational cross-sectional study, Bariatric surgery

## Abstract

**Purpose:**

To assess the occurrence and severity of SARS-CoV-2 infection/COVID-19, frequency of symptoms, clinical manifestations and behaviours in a sample of patients undergoing bariatric surgery (BS).

**Methods:**

The EPICOVID19-BS is an observational cross-sectional study conducted in Italy during the second wave of the COVID-19 pandemic (September 2021-February 2022). Patients with severe/extreme obesity undergoing BS were asked to complete an online multiple-choice questionnaire and to provide additional clinical information and blood biochemistry. Positive COVID-19 cases were defined by the combination of positive nasopharyngeal swab test results and/or positive serological test results. Sociodemographic, clinical and behavioural characteristics were compared between positive and negative COVID-19 cases.

**Results:**

A total of 745 participants were enrolled (mean age 44.5 ± 10.5 years SD, 78% female). The proportion of positive COVID-19 cases was 20.4%. They were more likely to be health care workers, to have close contacts with confirmed cases, to use anti-inflammatory drugs, to have immune system disorders, to have previous CMV infection, to have lower cholesterol levels and to have less metabolic syndrome than negative cases. Infected participants significantly increased their use of national health resources for minor health problems. The majority of participants experienced flu-like symptoms and taste and smell disturbances. Only 9.6% were hospitalised and none required intubation.

**Conclusions:**

Our results seem to support the evidence that patients undergoing BS have a low rate of severe SARS-CoV2. Further longitudinal studies in multiple obesity treatment centres are needed to more effectively monitor and control obesity in this specific population.

**Graphical Abstract:**

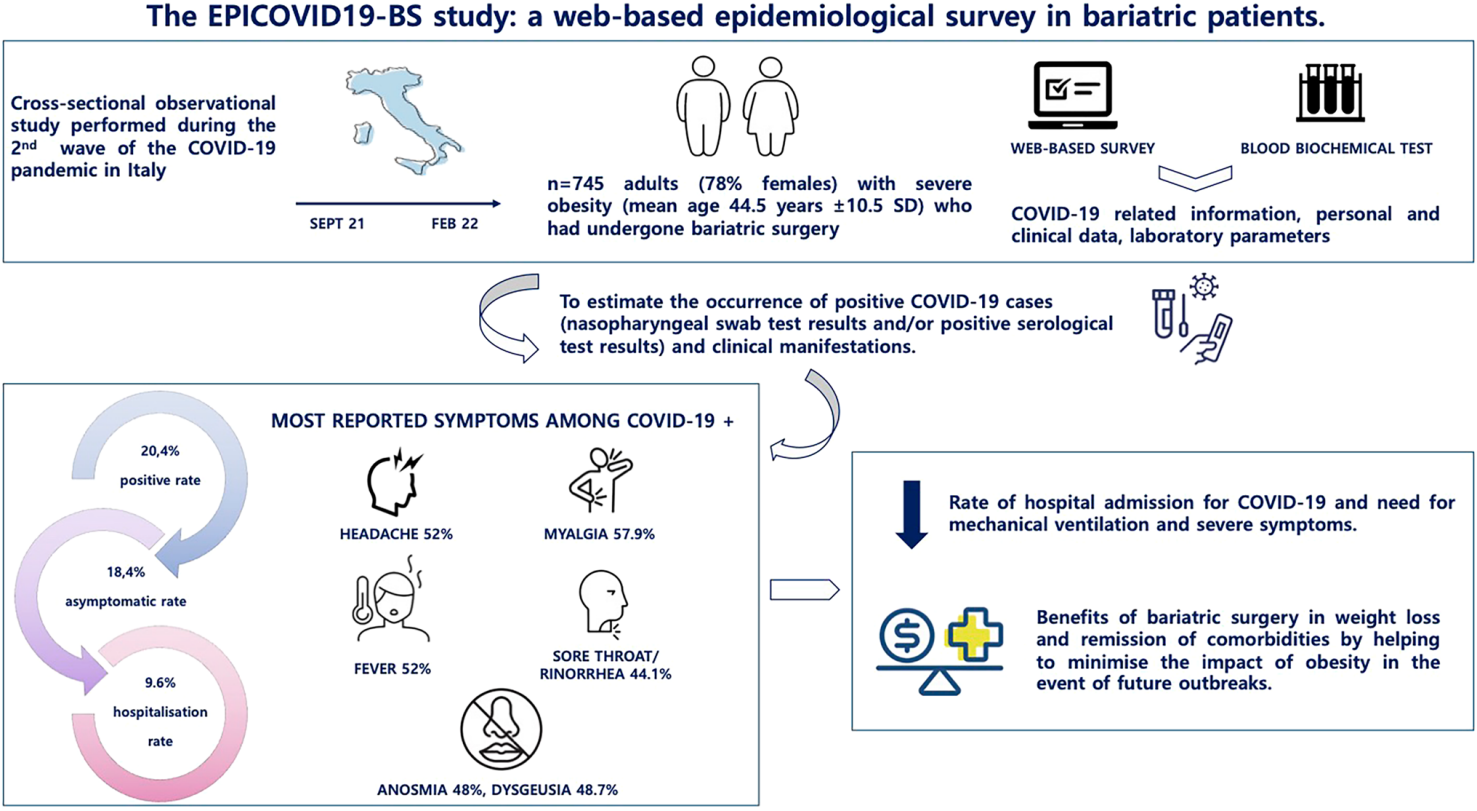

**Supplementary Information:**

The online version contains supplementary material available at 10.1007/s40618-024-02407-1.

## Introduction

Since the beginning of the coronavirus disease 2019 (COVID-19) pandemic, caused by the severe acute respiratory syndrome coronavirus 2 (SARS-CoV-2), several studies have investigated the risk factors for the infection and disease development [1]. Overweight or obesity has been recognised as an independent condition associated with significantly increased susceptibility to infection [2] and the development of COVID-19 complications and death [3–5]. Indeed, according to Lighter et al. [6], people with mild obesity (body mass index—BMI, between 30 and 34 kg/m^2^) under the age of 60 were 1.8 times more likely to be admitted to an intensive care unit (ICU) than those without obesity, and the risk was more than tripled for those with higher levels of obesity (BMI ≥ 35 kg/m^2^). In addition, patients with obesity tend to have a longer average hospital stay than those of normal weight (20.6 days vs. 16.0 days), which may indicate longer rehabilitation and discharge times, and they may also have a more severe clinical course of COVID-19 due to their higher viral load and slower antiviral response [7]. Furthermore, a retrospective study conducted in France, showed that intermittent ventilation increased with the degree of obesity and was higher in patients with a BMI > 35 kg/m^2^, a condition associated with lower survival rates [8]. Moreover, the likelihood of developing acute pneumonia increased 1327-fold with an obese phenotype, while the incidence of acute respiratory distress syndrome (ARDS), which causes acute and diffuse lung damage and subsequent respiratory failure, was significantly higher in the group with obesity than in the normal weight group (5.00% vs 0%) [9].

The pathogenetic mechanisms linking obesity to COVID-19 are diverse and not fully understood, but may involve several aspects such as respiratory dysfunction, dysregulated inflammation, SARS-CoV-2 entry, hyperglycemia and type 2 diabetes and adipokines [10–12].

Given the strong impact of obesity on the severity of the COVID-19, counteracting it through weight loss is an affordable strategy to implement from a public health perspective.

Bariatric surgery (BS) is currently considered the preferred treatment option for patients with a BMI of 35 or greater, and especially for those with a BMI of 40 or greater [13], because it can achieve a substantial and sustained weight loss, a reduction in comorbidities, an improvement in the obesity-related proinflammatory state, and survival benefits [14, 15]. Interestingly, data from the literature suggest that patients who have undergone BS appear to develop a less severe SARS-CoV-2 infection than those who have not, with a milder symptoms and clinical course [16], shorter duration of symptoms and hospitalisation duration, and lower rates of ICU and hospital admission and mortality [17–20]. However, no definitive conclusion can be drawn and data on the prevalence of the infection/disease and its clinical presentation in this specific population are scarce in Italy [16, 21], which was the third country worldwide in terms of total number of cases and the first in terms of total number of deaths [22]. Based on the above, we sought to perform a cross-sectional study to evaluate the occurrence of SARS-CoV-2 infection/COVID-19 in a sample of severe/extreme patients undergoing BS. The study also aims to estimate the severity of the infection, the frequency of symptoms, clinical manifestations and behaviours in patients who contracted the infection compared to those who did not.

## Materials and methods

### Study design, setting, and population

The EPICOVID19-BS is an Italian cross-sectional observational study carried out on a sample of adults aged between 18 and 69 years of both sexes with severe and extreme obesity (mean BMI 43.1, SD 6.0) undergoing BS. Patients were clinically followed up at the National Institute for Obesity Cure (INCO) of the Policlinico San Donato, IRCCS (Italy). Between 22 September 2021 and 23 February 2022, patients received an email invitation from the IRCCS with a personalised link asking them to complete the web-based EPICOVID19-BS questionnaire. The survey was implemented by the Institute of Biomedical Technologies–National Research Council (ITB-CNR) [23, 24] through the EUSurvey platform (https://ec.europa.eu/eusurvey/). Patients who did not complete the survey received monthly email reminders. To be included in the analysis, patients had to be able to understand the email instructions, complete the questionnaire, and give informed consent. Of the 1455 patients who received the link, 793 accessed the survey. Of these, 48 did not provide consent, leaving a final sample of 745 individuals for analysis.

### Variables collection, data transformation and analysis

The web questionnaire consisted of mandatory and closed questions, divided into different sections covering socio-demographic data, clinical assessment, COVID-19-related information, personal characteristics and health status, lifestyles and behaviors. To fully characterise the participants, in addition to the questionnaire responses, we collected laboratory parameters routinely performed on the patients by the IRCCS (only with the consent of the participants). *Socio-demographic* information included sex, age, educational level, occupational status, place of work, and category of work at risk of infection. The Townsend Deprivation Score (TDS) [25] was calculated as a proxy for individual-level deprivation by summing up, for each participant, the following variables (all dichotomised): unemployment, not owning the house in which he/she lives, not owning a family car, and overcrowding in the house (defined as the number of people living in the house greater than the number of rooms in the house, excluding kitchen and bathroom). The total score ranged from 0 to 4, with higher scores indicating greater deprivation (levels 3 and 4 were combined into level 3). *Clinical conditions* included diseases, medication use, other health conditions and vaccinations. The number of morbidities was defined by summing the total number of diseases and then grouped into none, one, and two or more. Altered glycaemia was defined as the presence of glycaemia greater than 110 mg/dl. Diabetes was classified as none (glycaemia less than 100 mg/dl and glycaemic haemoglobin less than 5.6% mmol/mol), pre-diabetes (glycaemia between 100 and 125 mg/dl and glycaemic haemoglobin between 5.7 and 6.4% mmol/mol) and diabetes (glycaemia greater than 126 mg/dl and glycaemic haemoglobin greater than 6.5% mmol/mol). Hypercholesterolaemia was defined as the presence of total cholesterol greater than 200 mg/dl and high-density lipoprotein (HDL) cholesterol less than 40 mg/dl in men or 50 mg/dl in women. Dyslipidaemia was defined as the presence of total cholesterol greater than 200 mg/dl, HDL cholesterol less than 40 mg/dl in men or 50 mg/dl in females, and triglycerides (TG) greater than 150 mg/dl. According to the NCEP ATP III definition, metabolic syndrome was defined as the presence of three or more of the following five criteria: waist circumference greater than 102 cm (men) or 88 cm (females), hypertension, fasting TG greater than 150 mg/dl, fasting HDL less than 40 mg/dl (males) or 50 mg/dl (females), and glycaemia greater than 100 mg/dl.

*COVID-19-related variables* included: contact with COVID-19 cases, self-isolation, nasopharyngeal swab (NPS) test, hospitalisation and medication, serological test (ST), anti-COVID-19 vaccination(s), and SARS-COV-2 infection-related symptoms. The primary outcome measure of the study was defined by combining the results of the NPS test and the serological test and classified as (i) no COVID-19 (a negative on the NPS test and a negative serological test); and COVID-19 (positive NPS test and/or positive results on the serological test). *Lifestyles and behaviours*. Sleep problems were measured using the Jeskin Sleep Scale (JSS) [26], with the total score ranged from 0 (no sleep problems) to 20. Perceived stress was measured using the 10-item Perceived Stress Scale (PSS) with the addition of five ad hoc items. Individual scores ranged from 0 to 40, with higher scores indicating greater perceived stress. Scores were categorised as follows: 0–13: low stress; 14–26: moderate stress; 27–40 high stress. Fear of infection for oneself or one’s relatives, fear about one’s own economic and employment situation, and fear about one’s relatives’ economic and employment situation were assessed with a short questionnaire developed ad hoc for this survey. The total score ranged from 0 to 16, with higher scores indicating greater fear. Individual feelings of being sufficiently informed about COVID-19 were dichotomised into a binary variable.

### Statistical analysis

Continuous variables were presented as mean and standard deviation (SD), and the categorical variables were presented as numbers and percentages. One-way analysis of variance and chi-squared test were used to compare the respondent characteristics according to COVID-19 status for continuous and categorical variables, respectively. The threshold of statistical significance for each test was set at a *p*-value of 0.05. All the statistical analyses were performed using SPSS (IBM Corp. Released, IBM SPSS Statistics version 25.0 Armonk, NY: IBM Corp.).

## Results

**Fig. 1:**
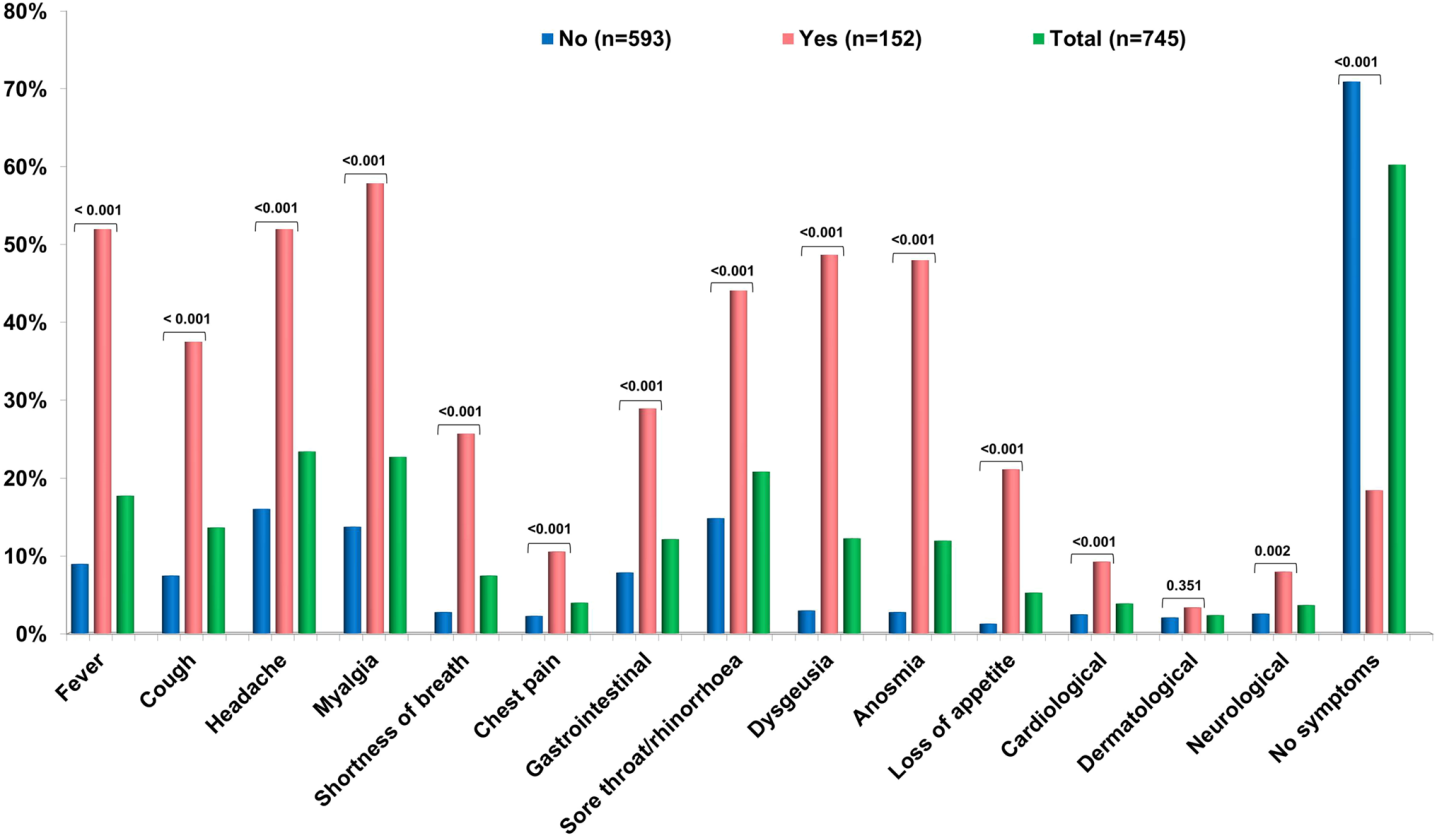
Distribution of symptoms by COVID-19 (n = 745)

**Fig. 2:**
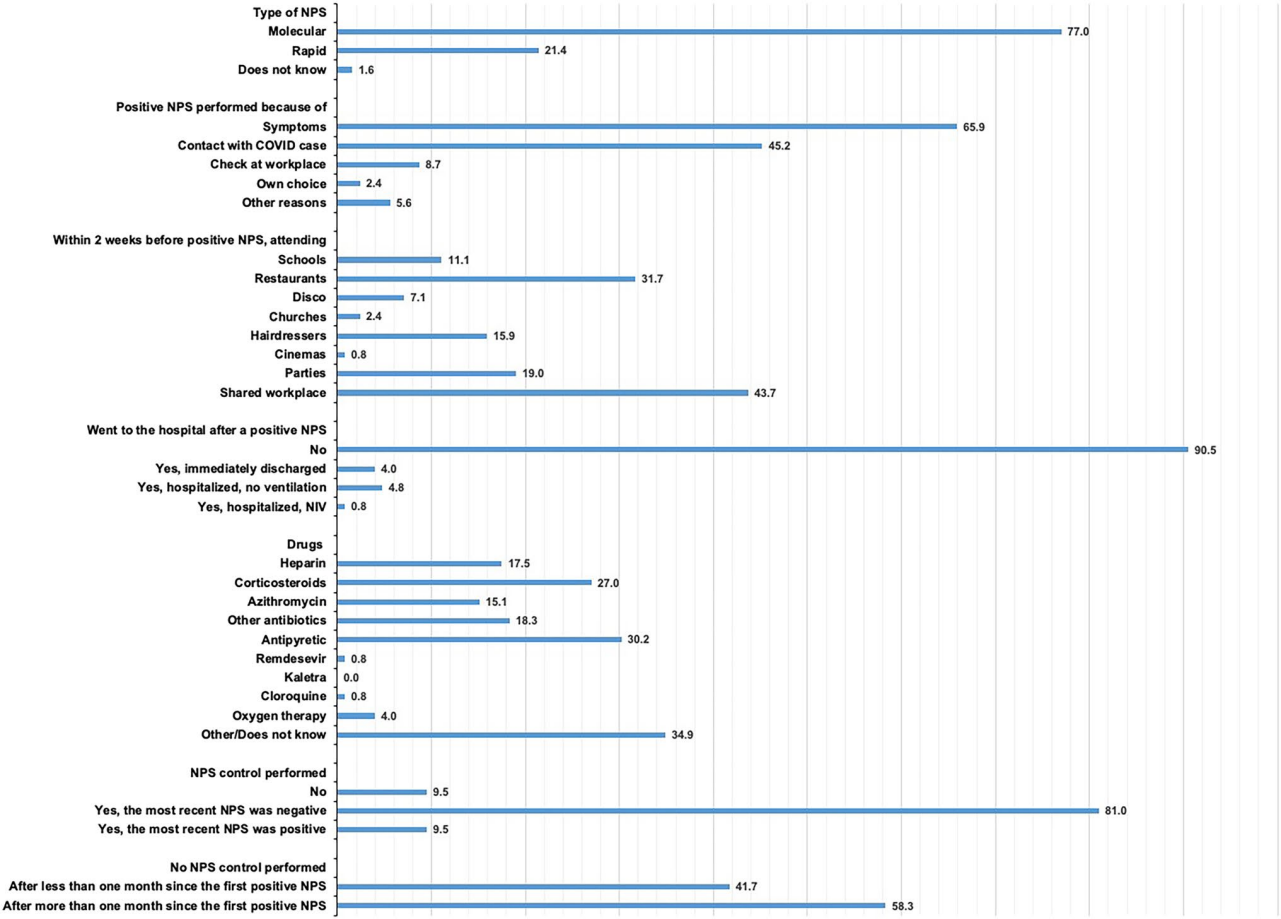
Positive Nasopharyngeal Swab, hospitalizations and drugs (n = 126)

This study presents descriptive analyses of the occurrence of SARS-CoV-2 infection/COVID-19, which was 20.4% (n = 152) in the 745 patients undergoing BS, with no statistically significant sex difference (17.3% in males vs 21.3% in females, data not shown). Table 1 shows the main sociodemographic characteristics of the study sample, which consisted of 581 females (78%). Participants were mostly aged between 18 and 65, with a mean age of 44.5 ± 10.5 years (SD). No significant differences were found between subjects with and without SARS-CoV-2 infection/COVID in terms of age, education level, employment status, place of work, or deprivation index. Healthcare workers were more likely to have SARS-CoV-2 than non-healthcare workers (25.9% vs 10.9%, *p*-value = 0.003). Table 2 shows that individuals with COVID-19 were more likely to have immune system disorders and cytomegalovirus infection, but less likely to have metabolic syndrome than those without COVID-19. A borderline difference in COVID-19 occurrence (*p*-value = 0.098) was observed for hypercholesterolaemia, with a higher prevalence in uninfected subjects. Over 80% of the study sample were taking medications to manage obesity-related pathologies, including anti-inflammatory (17.3%) and antihypertensive (16.8%) drugs, as well as vitamin D supplementation (52.6%) and multivitamin formulations (57.3%). Positive COVID-19 cases were more likely to take anti-inflammatory and anti-cancer drugs than controls. Pre-intervention haematochemical tests (Table 3) showed that total cholesterol was found to be higher in the negative subgroup and above the upper limit of the reference range. The majority of the sample reported no symptoms at all (60.3%) (Fig. 1 and Supplementary Table 1). Apart from dermatological symptoms (occurring in 3.3% of patients), a statistically significant higher proportion of COVID-19-positive subjects was observed for all other symptoms and not just for the pathognomonic taste and odour changes of SARS-CoV-2 infection. Most subjects experienced influenza-like symptoms, such as headache, myalgia, sore throat and fever. About one-fifth of the positive COVID-19 cases were asymptomatic. Table 4 shows COVID-19 related variables based on COVID-19 status. Of all respondents, 64.4% reported no close contact with positive cases and 63.2% had not been in quarantine or fiduciary self-isolation. The majority of healthy individuals reported no close contact with positive cases (70.3% compared with 41.4% of those who subsequently became ill), and the difference between the two groups was statistically significant. Approximately 95% of the sample underwent molecular testing for NPS with 126 COVID-19 cases (82.9%) testing positive at least once. In 68.2% of COVID-19 cases, 4 or more NPT tests were performed, compared to 36.5% of healthy subjects. The decision not to perform ST was significantly different between COVID-19 positives (48.7%) and COVID-19 negatives (74%). The majority of positive cases did not complete the full course of vaccination. Specifically, 29.6% of patients in this group had either never been vaccinated or had only received the first dose. In contrast, only 6.7% of negative patients did not complete the vaccination course, with 93.3% having completed it. Over two-thirds of the patient sample received the Pfizer-BioNTech vaccine. Figure 2 and Supplementary Table 2 show only the group of 126 subjects who tested positive for COVID-19. The primary reason for NPS testing with a positive result was the presence of COVID-19 symptoms (65.9%), followed by contact with a positive case (45.2%). Additionally, 43.7% of subjects reported sharing the workplace with a positive case within than 2 weeks prior to the positive NPS test result. Out of the 126 subjects, 12 (9.6%) were hospitalised following a positive NPS test, with an average hospital stay of 9.9 days. In the positive COVID-19 cases, 30.2% used antipyretics, followed by corticosteroids (27%) and generic antibiotics (18.3%). Five subjects required oxygen therapy, one of whom was admitted to the sub-intensive care unit and received non-invasive ventilation. In 81% of the 114 subjects tested, the NPS control was negative at the time of symptom resolution. The average time from the first positive swab to the last positive swab and from the first positive swab to the first negative swab was 27 and 20 days, respectively. Regarding a negative NPS test result (Table 5), it is noteworthy that 13% of those tested and yet infected took a negative NPS as a workplace infection control measure. This number increased to 48% among COVID-19 positive subjects, and the difference between cases and controls was statistically significant. There was no significant difference between the proportions of positive and negative COVID-19 respondents regarding their subjective perception of health status (Table 6). Participants reported feeling afraid for themselves, their loved ones or their economic/work situation, with a mean score of 7.6 on a scale of 0–16. In addition, 8.9% of respondents reported sleep disturbance. Stress levels were low in 54.1% of respondents, medium in 42.6% and high in 3.4%. The analysis shows that there was no significant difference in smoking or alcohol use or abuse between those with and without COVID-19 in the sample considered. The survey results indicate that 94.8% of respondents felt adequately informed about the new coronavirus, with no statistically significant differences between sick and healthy subjects. The final section of the questionnaire explored the impact of the pandemic and mandatory quarantine on various lifestyle habits (Table 7). The presence or absence of SARS-CoV-2 infection did not significantly affect eating habits since the start of the pandemic. Similarly, there were no differences between positive and negative COVID-19 cases in terms of sedentary time, physical activity and sleep time. These factors either increased, decreased or remained the same. Regarding access to national health resources for health problems, 9.9% of infected patients visited facilities for minor health problems, compared with 22.4% of those who did not contract the virus. Conversely, 20.4% of infected patients increased their visits, compared with 15.9% of healthy patients.

**Table 1:** Sociodemographic characteristics of the study sample by COVID-19 (n = 745)

	COVID-19			
	No	Yes	Total	p-value
Sex at birth				
Females	457 (77.1)	124 (81.6)	581 (78.0)	0.231
Males	136 (22.9)	28 (18.4)	164 (22.0)	
Prefers not to answer	0 (0.0)	0 (0.0)	0 (0.0)	
Age at surgery (yers)	44.7 ± 11.0	43.6 ± 10.2	44.5 ± 10.9	0.264
Class of age at surgery				
18–29	76 (12.8)	13 (8.6)	89 (11.9)	0.072
30–39	115 (19.4)	40 (26.3)	155 (20.8)	
40–49	175 (29.5)	54 (35.5)	229 (30.7)	
50–59	178 (30.0)	36 (23.7)	214 (28.7)	
60–69	49 (8.3)	9 (5.9)	58 (7.8)	
Educational level				
Low	163 (27.5)	49 (32.2)	212 (28.5)	0.139
Middle	330 (55.6)	71 (46.7)	401 (53.8)	
High	100 (16.9)	32 (21.1)	132 (17.7)	
Employment status since 1 Jun 2020				
Employed, stable position	375 (63.2)	102 (67.1)	477 (64.0)	0.534
Employed, occasional worker	37 (6.2)	10 (6.6)	47 (6.3)	
Temporary layoff	7 (1.2)	0 (0.0)	7 (0.9)	
Unemployed, as before 1 Jun 2020	51 (8.6)	11 (7.2)	62 (8.3)	
Unemployed, I lost my employment	27 (4.6)	5 (3.3)	32 (4.3)	
Student	18 (3.0)	2 (1.3)	20 (2.7)	
Retired	24 (4.0)	10 (6.6)	34 (4.6)	
Other	54 (9.1)	12 (7.9)	66 (8.9)	
Working at				
Workplace	321 (77.9)	89 (79.5)	410 (78.2)	0.610
Alternatively home and workplace	62 (15.0)	18 (16.1)	80 (15.3)	
Home	29 (7.0)	5 (4.5)	34 (6.5)	
Work category at risk				
No	232 (56.3)	47 (42.0)	279 (53.2)	0.003
Drivers, pilots, taxi drivers	9 (2.2)	4 (3.6)	13 (2.5)	
Staff working in beauty salons and hairdressers	5 (1.2)	1 (0.9)	6 (1.1)	
Personnel who work indoors with high turnout	78 (18.9)	19 (17.0)	97 (18.5)	
School staff	43 (10.4)	12 (10.7)	55 (10.5)	
Health staff	45 (10.9)	29 (25.9)	74 (14.1)	
Deprivation index^a^				
0	328 (55.3)	84 (55.3)	412 (55.3)	0.910
1	184 (31.0)	50 (32.9)	234 (31.4)	
2	70 (11.8)	15 (9.9)	85 (11.4)	
3	11 (1.9)	3 (2.0)	14 (1.9)	

^a^Townsend Deprivation Score: 0 (no deprivation) to 4 (high deprivation)

**Table 2:** Clinical characteristics of the study sample by COVID-19 (n = 745)

	COVID-19			
	No	Yes	Total	p-value
Lung diseases	41 (6.9)	14 (9.2)	55 (7.4)	0.334
Heart disease	13 (2.2)	3 (2.0)	16 (2.1)	0.868
Renal diseases	6 (1.0)	4 (2.6)	10 (1.3)	0.122
Diseases of the immune system	41 (6.9)	19 (12.5)	60 (8.1)	0.024
Oncological diseases	10 (1.7)	3 (2.0)	13 (1.7)	0.809
Gout	7 (1.2)	3 (2.0)	10 (1.3)	0.448
Neurological diseases	6 (1.0)	2 (1.3)	8 (1.1)	0.746
Cerebrovascular diseases	1 (0.2)	1 (0.7)	2 (0.3)	0.298
Hepatitis B	2 (0.3)	1 (0.7)	3 (0.4)	0.578
Hepatitis C	3 (0.5)	0 (0.0)	3 (0.4)	0.380
Other liver diseases	2 (0.3)	1 (0.7)	3 (0.4)	0.578
Depression and/or anxiety	49 (8.3)	16 (10.5)	65 (8.7)	0.378
Eating disorders	43 (7.3)	10 (6.6)	53 (7.1)	0.774
Anaemia	26 (4.4)	10 (6.6)	36 (4.8)	0.260
Hypertension disease and/or anti-hypertensive drugs	145 (24.5)	32 (21.1)	177 (23.8)	0.380
Altered glycemia	100 (16.8)	30 (19.7)	130 (17.4)	0.578
Dyslipidaemia	31 (5.2)	5 (3.3)	36 (4.8)	0.329
Hypercholesterolaemia	52 (8.8)	7 (4.6)	59 (7.9)	0.098
Diabetes				
No	204 (34.4)	57 (37.5)	261 (35.0)	0.568
Pre-diabetes	56 (9.4)	11 (7.2)	67 (9.0)	
Diabetes	22 (3.7)	7 (4.6)	29 (3.9)	
Hepatic steatosis	394 (66.4)	91 (59.9)	485 (65.1)	0.553
Metabolic Syndrome	163 (27.5)	31 (20.4)	194 (26.0)	0.024
No disease	322 (54.3)	75 (49.3)	397 (53.3)	0.274
N° of morbidities				
None	318 (53.6)	75 (49.3)	393 (52.8)	0.376
One	157 (26.5)	39 (25.7)	196 (26.3)	
Two or more	118 (19.9)	38 (25.0)	156 (20.9)	
Not self-sufficient	44 (7.4)	11 (7.2)	55 (7.4)	0.939
Pollen allergy	145 (24.5)	38 (25.0)	183 (24.6)	0.889
Food allergy	94 (15.9)	26 (17.1)	120 (16.1)	0.708
Recurrent Herpes	74 (12.5)	25 (16.4)	99 (13.3)	0.198
CMV infection	13 (2.2)	12 (7.9)	25 (3.4)	< 0.001
HPV infection	24 (4.0)	2 (1.3)	26 (3.5)	0.102
*Medication use*				
Aspirin	30 (5.1)	8 (5.3)	38 (5.1)	0.919
Anti-hypertensive	101 (17.0)	24 (15.8)	125 (16.8)	0.715
Cholesterol treatment drugs	36 (6.1)	9 (5.9)	45 (6.0)	0.945
Diabetes medicines	26 (4.4)	6 (3.9)	32 (4.3)	0.813
Anti-cancer drugs	0 (0.0)	2 (1.3)	2 (0.3)	0.005
Thyroid drugs	67 (11.3)	17 (11.2)	84 (11.3)	0.968
Anti-inflammatory drugs	93 (15.7)	36 (23.7)	129 (17.3)	0.020
Anxiety medications and/or sedatives	27 (4.6)	9 (5.9)	36 (4.8)	0.483
Anti-depressants	53 (8.9)	12 (7.9)	65 (8.7)	0.684
Oral cortisone	9 (1.5)	3 (2.0)	12 (1.6)	0.690
Cortisone by inhalation	17 (2.9)	5 (3.3)	22 (3.0)	0.784
Vitamin D	306 (51.6)	86 (56.6)	392 (52.6)	0.273
Multivitamins	331 (55.8)	96 (63.2)	427 (57.3)	0.103
No drugs	115 (19.4)	23 (15.1)	138 (18.5)	0.228

**Table 3:** Pre-intervention laboratory parameters by COVID-19 (n = 745)

	COVID-19			
	No	Yes	Total	p-value
Leukocytes	9.7 ± 8.0	8.9 ± 7.6	9.5 ± 7.9	0.387
Erythrocytes	8. 0 ± 36.6	5.1 ± 1.5	7.4 ± 30.8	0.383
Haemoglobin	16.5 ± 16.2	17.8 ± 21.0	16.8 ± 17.3	0.507
Hematocrit	43.9 ± 8.7	42.8 ± 8.6	43.7 ± 8.7	0.267
Mean corpuscular Volume	84.3 ± 12.6	83.6 ± 11.5	84.2 ± 12.3	0.609
Mean corpuscular haemoglobin	31.2 ± 27.9	28.4 ± 3.2	30.6 ± 24.8	0.307
Mean corpuscular haemoglobin concentration	38.0 ± 43.4	44.6 ± 60.8	39.4 ± 47.6	0.214
Red Cell Distribution Width	26.8 ± 15.3	25.6 ± 14.8	26.5 ± 15.2	0.525
Platelets	279.7 ± 72.1	273.3 ± 79.9	278.3 ± 73.9	0.428
Mean platelet volume	12.9 ± 14.9	10.6 ± 6.3	12.4 ± 13.5	0.194
Neutrophil granulocytes	6.6 ± 8.4	6.1 ± 6.6	6.4 ± 8.1	0.578
Lymphocytes	3.2 ± 4.6	2.9 ± 3.7	3.1 ± 4.4	0.559
Monocytes	0.8 ± 1.6	0.6 ± 0.7	0.8 ± 1.4	0.250
Eosyophilic granulocytes	0.5 ± 2.8	0.3 ± 0.9	0.5 ± 2.5	0.463
Neutrophil granulocytes A	0.5 ± 3.2	0.1 ± 0.2	0.4 ± 2.8	0.255
Creatinemia	0.9 ± 1.9	0.8 ± 0.3	0.9 ± 1.7	0.440
Aspartate Aminotransferase- AST	24.6 ± 12.9	22.5 ± 8.0	24.2 ± 12.1	0.153
Alanine Aminotransferase-ALT	31.7 ± 21.2	31.4 ± 27.5	31.7 ± 22.6	0.896
Gamma Glutamyl Transferase	27.9 ± 37.7	39.0 ± 51.8	30.1 ± 41.0	0.160
Total Cholesterol	204.5 ± 40.8	191.8 ± 35.2	201.9 ± 40.0	0.006
HDL cholesterol	52.4 ± 12.8	54.8 ± 14.2	52.9 ± 13.1	0.125
Triglycerides	143.7 ± 86.0	125.3 ± 73.5	140.0 ± 83.9	0.061
Glycemic changes	106.8 ± 28.5	106.1 ± 27.1	106.7 ± 28.2	0.806
Glycated haemoglobin	7.7 ± 9.7	6.1 ± 3.5	7.4 ± 8.8	0.153
Insulinaemia	24.1 ± 15.7	24.4 ± 24.2	24.1 ± 17.4	0.919
Uricemia	5.5 ± 2.0	5.8 ± 2.5	5.6 ± 2.1	0.394
Vitamin D	21.8 ± 14.7	23.5 ± 13.9	22.2 ± 14.5	0.337
Ionized calcium	2.6 ± 1.8	2.5 ± 1.9	2.6 ± 1.8	0.789
Parathyroid hormone-PTH	61.4 ± 33.4	57.7 ± 23.8	60.6 ± 31.6	0.352
Thyroid-stimulating hormone reflex-TSH	2.5 ± 2.0	2.6 ± 1.7	2.5 ± 1.9	0.601
Thyroperoxidase antibodies-AbTPO ≥ 60 UI/mL				
Negative	242 (90.6)	66 (90.4)	308 (90.6)	0.953
Positive	25 (9.4)	7 (9.6)	32 (9.4)

**Table 4:** COVID-19 related variables by COVID-19 (n = 745)

	COVID-19			
	No	Yes	Total	p-value
Close contact with confirmed COVID-19 cases				
No	417 (70.3)	63 (41.4)	480 (64.4)	< 0.001
Yes, while wearing a face mask	85 (14.3)	37 (24.3)	122 (16.4)	
Yes, at least once without wearing a face mask	91 (15.3)	52 (34.2)	143 (19.2)	
Quarantine or fiduciary isolation				
Never	451 (76.1)	20 (13.2)	471 (63.2)	< 0.001
Once	119 (20.1)	110 (72.4)	229 (30.7)	
More than once	23 (3.9)	22 (14.5)	45 (6.0)	
A molecular test for SARS-CoV-2 performed				
No	39 (6.6)	1 (0.7)	40 (5.4)	< 0.001
Yes, always with a negative result	554 (93.4)	25 (16.4)	579 (77.7)	
Yes, with a positive result at least once	0 (0.0)	126 (82.9)	126 (16.9)	< 0.001
Number of NPS				
1	115 (20.8)	3 (2.0)	118 (16.7)	
2	131 (23.6)	23 (15.2)	154 (21.8)	
3	106 (19.1)	22 (14.6)	128 (18.2)	
4 or more	202 (36.5)	103 (68.2)	305 (43.3)	
Serological test for SARS-CoV-2 performed				
No	439 (74.0)	74 (48.7)	513 (68.9)	< 0.001
Yes, always with a negative result	154 (26.0)	31 (20.4)	185 (24.8)	
Yes, with a positive result at least once	–	47 (30.9)	47 (6.3)	
Anti-COVID-19 vaccination				
Not performed or only one dose	40 (6.7)	45 (29.6)	85 (11.4)	< 0.001
Both doses	553 (93.3)	107 (70.4)	660 (88.6)	
Type of vaccination				
Pfizer-BioNtech	444 (78.6)	114 (77.6)	558 (78.4)	0.737
AstraZeneca	34 (6.0)	6 (4.1)	40 (5.6)	
Johnson & Johnson	10 (1.8)	4 (2.7)	14 (2.0)	
Moderna	76 (13.5)	23 (15.6)	99 (13.9)	
Don't know	1 (0.2)	0 (0.0)	1 (0.1)	
If not vaccinated, is going to receive Covid-19 vaccination				
No	8 (28.6)	3 (60.0)	11 (33.3)	0.410
Probably not, but more information is needed	7 (25.0)	0 (0.0)	7 (21.2)	
Not able to answer now	4 (14.3)	1 (20.0)	5 (15.2)	
Probably yes, but more information is needed	3 (10.7)	1 (20.0)	4 (12.1)	
Yes	6 (21.4)	0 (0.0)	6 (18.2)

**Table 5:** Negative Nasopharyngeal Swab and exposures to the risk of infection (n = 579)

	COVID-19			
	No	Yes	Total	p-value
Type of NPS				
Molecular	294 (53.1)	16 (64.0)	310 (53.5)	0.332
Rapid	226 (40.8)	9 (36.0)	235 (40.6)	
Does not know	34 (6.1)	0 (0.0)	34 (5.9)	
Negative NPS done because of				
Symptoms	39 (7.0)	3 (12.0)	42 (7.3)	0.350
Contact with COVID case	70 (12.6)	6 (24.0)	76 (13.1)	0.100
Check at workplace	73 (13.2)	12 (48.0)	85 (14.7)	< 0.001
Own choice	85 (15.3)	1 (4.0)	86 (14.9)	0.119
Other reasons	330 (59.6)	5 (20.0)	335 (57.9)	< 0.001
Number of days since onset of symptoms to negative NPS	1.7 ± 0.6	2.0 ± 1.7	1.7 ± 0.7	0.486
Within 2 weeks before negative NPS, attending				
Schools	69 (12.5)	6 (24.0)	75 (13.0)	0.093
Restaurants	222 (40.1)	11 (44.0)	233 (40.2)	0.695
Disco	35 (6.3)	3 (12.0)	38 (6.6)	0.262
Churches	31 (5.6)	0 (0.0)	31 (5.4)	0.224
Hairdressers	128 (23.1)	7 (28.0)	135 (23.3)	0.571
Cinemas	15 (2.7)	1 (4.0)	16 (2.8)	0.700
Parties	142 (25.6)	9 (36.0)	151 (26.1)	0.248
Means of transport	64 (11.6)	2 (8.0)	66 (11.4)	0.585
Shared work place	251 (45.3)	16 (64.0)	267 (46.1)	0.067

**Table 6:** Personal characteristics and health status by COVID-19 (n = 745)

	COVID-19			
	No	Yes	Total	p-value
Perceived health status				
Very bad or bad	8 (1.3)	3 (2.0)	11 (1.5)	0.120
Adequate	100 (16.9)	36 (23.7)	136 (18.3)	
Good or very good	485 (81.8)	113 (74.3)	598 (80.3)	
Fear score	7.7 ± 4.2	7.4 ± 4.3	7.6 ± 4.2	0.430
Sleep disorders	54 (9.1)	12 (7.9)	66 (8.9)	0.639
Perceived stress				
Low	318 (53.6)	85 (55.9)	403 (54.1)	0.790
Moderate	254 (42.8)	63 (41.4)	317 (42.6)	
High	21 (3.5)	4 (2.6)	25 (3.4)	
During last year, the frequency of alcoholic beverages between meals				
Never	311 (52.4)	72 (47.4)	383 (51.4)	0.103
≤ once a week	239 (40.3)	73 (48.0)	312 (41.9)	
2–3 times a week	28 (4.7)	7 (4.6)	35 (4.7)	
More than 4 times a week	15 (2.5)	0 (0.0)	15 (2.0)	
Smoking habit				
Never	302 (50.9)	85 (55.9)	387 (51.9)	0.484
Former	142 (23.9)	35 (23.0)	177 (23.8)	
Current	149 (25.1)	32 (21.1)	181 (24.3)	
If smokers, use electronic cigarettes				
No	72 (48.3)	14 (43.8)	86 (47.5)	0.727
Yes, not exclusively	53 (35.6)	11 (34.4)	64 (35.4)	
Yes, exclusively	24 (16.1)	7 (21.9)	31 (17.1)	
Passive smoking				
No	424 (71.5)	113 (74.3)	537 (72.1)	0.281
Yes, one person	100 (16.9)	18 (11.8)	118 (15.8)	
Yes, more than one person	69 (11.6)	21 (13.8)	90 (12.1)	
F eel sufficiently informed about COVID-19	565 (95.3)	141 (92.8)	706 (94.8)	0.214

**Table 7:** Changes in lifestyles and in access to national health facilities by COVID-19 (n = 745)

	COVID-19			
	No	Yes	Total	p-value
Since the pandemic started, changes in				
Snacking habits				
Not present, or not applicable	70 (11.8)	15 (9.9)	85 (11.4)	0.491
Decreased	84 (14.2)	17 (11.2)	101 (13.6)	
Unchanged	265 (44.7)	67 (44.1)	332 (44.6)	
Increased	174 (29.3)	53 (34.9)	227 (30.5)	
Skipping meals				
Not present, or not applicable	221 (37.3)	49 (32.2)	270 (36.2)	0.619
Decreased	88 (14.8)	23 (15.1)	111 (14.9)	
Unchanged	232 (39.1)	63 (41.4)	295 (39.6)	
Increased	52 (8.8)	17 (11.2)	69 (9.3)	
Daily consumption of fruit/vegetables				
Not present, or not applicable	32 (5.4)	8 (5.3)	40 (5.4)	0.854
Decreased	88 (14.8)	23 (15.1)	111 (14.9)	
Unchanged	336 (56.7)	81 (53.3)	417 (56.0)	
Increased	137 (23.1)	40 (26.3)	177 (23.8)	
Habit of a balanced diet				
Not present, or not applicable	29 (4.9)	9 (5.9)	38 (5.1)	0.922
Decreased	49 (8.3)	14 (9.2)	63 (8.5)	
Unchanged	297 (50.1)	73 (48.0)	370 (49.7)	
Increased	218 (36.8)	56 (36.8)	274 (36.8)	
Consumption of sweet foods				
Not present, or not applicable	107 (18.0)	25 (16.4)	132 (17.7)	0.186
Decreased	253 (42.7)	54 (35.5)	307 (41.2)	
Unchanged	163 (27.5)	47 (30.9)	210 (28.2)	
Increased	70 (11.8)	26 (17.1)	96 (12.9)	
Consumption of fried or fast foods				
Not present, or not applicable	194 (32.7)	49 (32.2)	243 (32.6)	0.184
Decreased	245 (41.3)	51 (33.6)	296 (39.7)	
Unchanged	123 (20.7)	41 (27.0)	164 (22.0)	
Increased	31 (5.2)	11 (7.2)	42 (5.6)	
Alcohol consumption				
Not present, or not applicable	309 (52.1)	78 (51.3)	387 (51.9)	0.749
Decreased	139 (23.4)	31 (20.4)	170 (22.8)	
Unchanged	132 (22.3)	39 (25.7)	171 (23.0)	
Increased	13 (2.2)	4 (2.6)	17 (2.3)	
Physical activity				
Not present, or not applicable	71 (12.0)	27 (17.8)	98 (13.2)	0.249
Decreased	100 (16.9)	26 (17.1)	126 (16.9)	
Unchanged	190 (32.0)	41 (27.0)	231 (31.0)	
Increased	232 (39.1)	58 (38.2)	290 (38.9)	
Sedentariness				
Not present, or not applicable	42 (7.1)	17 (11.2)	59 (7.9)	0.248
Decreased	122 (20.6)	25 (16.4)	147 (19.7)	
Unchanged	253 (42.7)	69 (45.4)	322 (43.2)	
Increased	176 (29.7)	41 (27.0)	217 (29.1)	
Hours of sleep				
Not present, or not applicable	30 (5.1)	8 (5.3)	38 (5.1)	0.884
Decreased	135 (22.8)	32 (21.1)	167 (22.4)	
Unchanged	359 (60.5)	91 (59.9)	450 (60.4)	
Increased	69 (11.6)	21 (13.8)	90 (12.1)	
Accessing the national health resources for mild problems of health				
Not present, or not applicable	74 (12.5)	25 (16.4)	99 (13.3)	0.005
Decreased	133 (22.4)	15 (9.9)	148 (19.9)	
Unchanged	292 (49.2)	81 (53.3)	373 (50.1)	
Increased	94 (15.9)	31 (20.4)	125 (16.8)	
Accessing the national health resources for serious				
Not present, or not applicable	227 (38.3)	55 (36.2)	282 (37.9)	0.222
Decreased	82 (13.8)	13 (8.6)	95 (12.8)	
Unchanged	205 (34.6)	63 (41.4)	268 (36.0)	
Increased	79 (13.3)	21 (13.8)	100 (13.4)	
Contacting the general practitionnaire				
Not present, or not applicable	54 (9.1)	12 (7.9)	66 (8.9)	0.093
Decreased	119 (20.1)	21 (13.8)	140 (18.8)	
Unchanged	336 (56.7)	87 (57.2)	423 (56.8)	
Increased	84 (14.2)	32 (21.1)	116 (15.6)	

## Discussion

The EPICOVID19-BS epidemiological survey is a continuation of the EPICOVID-19 study, which targeted individuals aged 18 years and older residing in Italy and was led by the same working group. EPICOVID19 included a phase I epidemiological survey to determine the prevalence of suspected SARS-CoV-2 infection and associated factors in a sample of 201,121 adults residing in Italy during the first wave of the pandemic (April–May 2020) [23]. In addition, a phase II follow-up study of 43,473 individuals was conducted in Italy in January–February 2021 [24].

In the present study, performed from September 2021 to February 2022, the rate of positive COVID-19 cases was 20.4%, which is higher than the rate of 13.2% found in the follow-up EPICOVID19 study performed in the general population, as expected [23]. Few studies have reported the prevalence of positive NPS/COVID-19 in the BS population. In a retrospective observational cohort study of 236 Iranian patient with severe obesity following (surgical group) or candidates (nonsurgical group) for BS contacted by questionnaire between November 2020 and March 2021, the incidence of probable COVID-19 was reported to be 20.6% in the surgical group and 26.08% in the non-surgical groups [19]. In the study by Romero-Velez and colleagues, 190 patients with severe obesity are at high risk of severe disease secondary to COVID-19 were contacted by telephone from January 2020 to March 2020, and those who underwent bariatric surgery during the development of the pandemic reported COVID-19 compatible symptoms 30 days after surgery at a rate of 10.7% and 3.4% went on to test positive [20]. In Italy, Marchesi et al. conducted a study of 594 patients from various Italian bariatric centres who underwent a telephone survey from April to August 2020; the authors reported that the rate of probable cases was 14.4% in the operated group and 23.7% in the candidate group [16]. Marinari et al. performed a structured interview in January–February 2020 with 840 patients who had undergone surgery before the outbreak and found only 5 cases of infection (0.6%) without mortality [21].

Regarding the sociodemographic characteristics of the sample, we enrolled more females than males with an average age in the 40–49 age group, and a majority of participants with a medium level of education. This sex disparity may be partly due to the fact that females are more likely than males to undergo weight loss surgery, with recent global reports suggesting that 70% of patients undergoing bariatric surgery are females [27]. No statistically significant differences between sex, age and educational level in the occurrence of COVID-19 were observed.

Instead, we found a significant difference in terms of occupational risk categories, with 29 (39%) of 74 patients working in the healthcare sector contracting SARS-CoV-2. This is consistent with the scientific literature on occupational risk factors: according to a meta-analysis of 97 studies by Gholami and colleagues, healthcare workers reported the highest rate of infection [28].

The authors of the phase I EPICOVID19 study took into account the number of comorbidities without separating the reported chronic diseases. As these subjects were patients undergoing BS, it is consistent that the types and frequencies of pathologies in EPICOVID19-BS differ from those in the study by Adorni F. et al. (2020): the most common chronic diseases were hypertension (23.8% of the enrolled subjects), depression and/or anxiety (8.7% of the subjects), dyslipidaemia (4.8% of the subjects), diabetes (3.9% of the subjects) and diseases of the immune system (8.1% of the total subjects, of which 12.5% of the total positive cases. Obesity is known to reduce self-tolerance mechanisms by promoting a pro-inflammatory environment for the development of autoimmune diseases, such as Hashimoto's thyroiditis; the aetiological agent of COVID-19, likewise, may also generate a cytokine storm, and in some cases may even be a disease trigger or precipitating factor. However, the relationship between autoimmune disease and susceptibility to SARS-CoV-2 is unclear [29]. When information on adverse environmental, food or pharmacological allergic reactions and previous infections were taken into account, previous cytomegalovirus infection appeared to increase the probability of contracting SARS-CoV-2. This observation is supported by the results of a retrospective study investigating the CMV serostatus of non-geriatric patients admitted to the ICU for COVID-19, which found that the CMV seropositivity, in contrast to herpes simplex virus seropositivity used as a control, could be a strong marker for detecting a higher risk of COVID-19 in younger subjects in the absence of other diagnosed comorbidities [30].

The use of medication was almost universal, but the following stood out: anti-inflammatory drugs, taken as needed, mainly to relieve joint pain and chronic low back pain, followed by anti-hypertensive drugs to control the main cardiovascular complication of obesity; more than half of the sample, suffering from vitamin D insufficiency or deficiency, typical of obesity, reported taking specific supplements instead, while 57.3% of them took multivitamins to correct multiple deficiencies. We found that of the 129 subjects who reported taking anti-inflammatory drugs, about 24% contracted SARS-CoV-2 infection. Several pharmacoepidemiologic studies confirm that exposure to non-steroidal anti-inflammatory drugs in viral or bacterial lung infections may increase the risk of severe complications, such as pleural empyema, necrotising pneumonia, or lung abscess [31].

Total cholesterol at or above the upper limit of normal, but without a diagnosis of dyslipidaemia, appeared to be lower in positive COVID-19 cases, as was the presence of metabolic syndrome. Indeed, we also found that of 194 patients diagnosed with metabolic syndrome as defined by the NCEP guidelines (ATP III) [32], 84% did not have COVID-19, which is not reflected in the literature where, for example, in a sample of 8885 subjects, the cumulative incidence of COVID-19 was found to be higher in patients with metabolic syndrome [33]. Lipidomic studies have shown that lipids are essential for viruses to cross host cell membranes and that enveloped coronaviruses in particular can alter intracellular metabolism and signaling to facilitate their replication. SARS-CoV-2 targets lipid droplets and exploits endosomes to make copies of itself; this causes the endoplasmic reticulum to produce misfolded proteins that trigger a chain reaction leading to downstream stimulation of sterol regulatory-element binding protein-1 (SREBP-1), which transcribes lipid down-regulation genes. The result is the most common lipid profile alterations reported in the literature, namely a decrease in total cholesterol, Apolipoprotein A1 levels, and a concomitant increase in circulating TG levels. Furthermore, the hyperinflammatory state affects several lipid biosynthetic pathways, and the more HDL-C and its major apolipoprotein are reduced, the greater the severity of disease, mortality and levels of inflammatory markers [34]. In addition to the lipid picture, it is evident that the sample reference population is also partially affected by pre-diabetes or overt diabetes, a condition that has not been shown to be negatively or positively associated with COVID-19 incidence and prognosis, although studies have reported that both glycaemia and glycated haemoglobin are significantly higher in affected individuals [35].

Compared to the study by Adorni F. et al. (2020) [23], where the asymptomatic rate was 7.7%, we found an asymptomatic rate of 18.4% in our study. Most subjects experienced flu-like symptoms, such as headache, myalgia, sore throat/rhinorrhoea and fever in addition to loss of smell and taste, a pathognomonic feature of earlier waves of SARS-CoV-2 infection and suggested as key symptoms of mild-to-moderate COVID-19 patients [36].

As evidence of the insidious mode of transmission of the virus through the air or by close direct contact with droplets, as highlighted in the scientific literature, a much higher frequency of subjects who fell ill reported having had "close contact" with confirmed COVID-19 cases, one third of the COVID-19 positive group at least once without wearing a mask to protect the oronasal mucosa. However, the open awareness of the high level of contagiousness, which also exploited a conspicuous proportion of asymptomatic subjects as unwitting carriers of the disease, was not sufficient to induce subjects without suspicious symptoms to perform a precautionary nasopharyngeal swab, which later proved negative, after contact with confirmed COVID-19 cases. The strong ego-syntonic psychopathological correlate supporting and maintaining their state of obesity was manifested in an overestimation of their health status, which ended in their low perception of anxiety and stress due to the new pandemic challenge they had to face. It is now well established that each BMI point above the range defined as "normal weight" increases the rate of hospitalisation, use of intensive care, illness and length of convalescence: however, our study showed a low percentage of hospitalisations among the COVID-19 positive (9.6%), of whom five subjects required oxygen therapy and one of them was admitted to the sub-intensive care unit and received non-invasive ventilation, and none were intubated. For example, Lighter et al. reported 29% and 22% of acute admissions due to COVID-19 and 23% and 33% of ICU admissions in US patients aged < 60 years with a BMI between 30 and 34 and greater than 35, respectively [6]. Our findings were consistent with the results from two recent meta-analyses involving 150,848 [17] and 151,475 [18] patients, respectively, which showed that BS is associated with a reduced severity of COVID-19 infection, as evidenced by a reduced risk of mortality, hospital and ICU admission, mechanical ventilation, and shorter hospital stay in the surgical group of patients with obesity after SARS-CoV-2 infection compared with the non-operative group.

The contribution of obesity to the severity of COVID-19 can be explained in several ways [4]. Adipose tissue has higher levels of ACE2 angiotensin-converting enzyme 2 (ACE2) receptors, the key entry mechanism of SARS-CoV-2, than human lung, a major target tissue affected by viral infection. In patients with obesity, adipose tissue grows by hyperplasia, resulting in an increased number of cells expressing ACE2, increasing the likelihood of SARS-CoV-2 entry [37]. Obesity also affects respiratory function through several mechanisms, including mechanical changes due to fat deposition on the chest wall, diaphragm, and upper airways, which can lead to restrictive lung damage [38]. In addition, individuals with obesity experience persistent chronic low-grade systemic inflammation and disproportionate adipocyte volume resulting in low blood perfusion. Adipose tissue hypoxia increases pro-inflammatory signals, which in turn cause dysregulation of the immune response. COVID-19 cases with obesity are more likely to develop critical symptoms due to the well-known 'cytokine storm' [39]. Weight loss and long-term reduction in adipose tissue as a result of BS might help to reduce the number of ACE2-expressing cells, improve respiratory function [40] and reduce inflammatory markers such as C-reactive protein (CRP) and IL-6 [41].

Regarding lifestyle (smoking, alcohol, dietary habits), questionnaire responses showed no significant differences between positive and negative COVID-19 cases. Overall, most enrolled subjects reported light to moderate alcohol consumption and only ¼ were current smokers. Given the enforced confinement to the home during the first wave of the pandemic, there was no change in physical activity, sedentary behaviour, sleep duration or eating behaviours typical of individuals with severe obesity, such as snacking, skipping meals and eating sweet, fried or fast food. We must therefore give the benefit of the doubt and assume that the patient with obesity who presents to a bariatric surgery unit either has a misconception about his disease and his bad habits, or is well aware of them but is reluctant and ashamed to admit his dysfunctional behaviour. Since the beginning of the pandemic, there has been a significant difference in the frequency of visits to national health facilities for non-severe health problems between COVID-19 infected and negative patients. In the present study, we observed that in the former, it was only 9.9% compared with 22.4% of those who did not become ill; conversely, in the latter, it was 20.4% of those who became ill compared with 15.9% of those who did not, apparently because of the presence of typical symptoms or a strong suspicion of positivity; the latter either because of previous close contact with other COVID-19-positive individuals or because at least one of them had a positive molecular swab result. During the study period, hospitals were facing a crisis of limited human resources, with entire wards, operating theatres and outpatient clinics being reserved for COVID-19 emergencies. This, combined with the fear of infection, led to a reduction in general practitioner visits, outpatient visits, intensive care, pathology and oncology screening, with the result that the frailest population, including the overweight and obese, ended up with a chronic disease that was not previously present.

### Limitations and strengths

There are several limitations that need to be considered. Due to the cross-sectional and observational design, it is not possible to draw causal inferences. In addition, the study is voluntary, which may affect the generalisability of the results. In fact, some of the characteristics of the sample may not be representative of the Italian adult population undergoing BS, thus comparison with other cohorts should be made with caution. Another limitation of our study is the use of patient self-report data, which may have introduced measurement error and recall bias. This may have led to misclassification of participants' COVID-19 status or exposures. However, it is reasonable to assume that non-differential misclassification may have occurred, where the likelihood of misclassification of exposure is independent of disease status and vice-versa, increasing the similarity between the exposed and unexposed groups. A future well-designed longitudinal prospective cohort study or randomised controlled clinical trial, including radiographs and chest CT scans at hospital admission and discharge, and postoperative follow-up of enrolled patients, is needed to more accurately assess exposure and clinical risk in this vulnerable population subgroup. Nevertheless, the study has notable strengths. First, the study included a large sample of patients undergoing bariatric surgery in Italy. Second, although the data were self-reported, the entire sample underwent an NPS or ST, providing a snapshot of the positivity rate in this specific population for which data are scarce. Third, the sample was well characterised by the extensive collection of socio-demographic, behavioural and psychological data through the web survey, combined with the previously collected detailed clinical information and laboratory parameters. Fourthly, the use of a web-based survey to collect data can overcome the higher costs associated with active follow-up or interviewer bias typical of telephone survey, as it is inexpensive and can rapidly involve a large number of people regardless of geographical distance.

## Conclusions

Our results seem to support the evidence that patients who undergo bariatric surgery have a low rate of hospital admission for COVID-19 and need for mechanical ventilation, as well as less severe symptoms, and that this is partly justified by the multiple benefits in terms of weight loss and remission of comorbidities that surgery can offer if the patient has been well selected by a multidisciplinary team and has good compliance with dietary and lifestyle recommendations. Therefore, due to the direct and indirect costs of obesity and COVID-19, weight loss through bariatric surgery can be considered the best strategy, combining the possibility of providing the best care to patients with obesity with the possibility of economic savings [42] by helping to minimise the impact of the disease in the event of future outbreaks.

## Supplementary Information

Below is the link to the electronic supplementary material.

Supplementary file1 (DOCX 19 KB)

## Data Availability

The datasets generated during and/or analysed during the current study are available from the corresponding author upon reasonable request.

## Abbreviations

COVID-19: Coronavirus Disease 2019
SARS-CoV-2: Severe Acute Respiratory Syndrome COronaVirus 2
ICU: Intensive care unit
BMI: Body mass index
ARDS: Severe acute respiratory syndrome
TDS: Townsend Deprivation Score
HDL: High-density lipoprotein
TG: Triglycerides
NCEP: National Cholesterol Education Program
ATP: Adult Treatment Panel
NPS: Nasopharyngeal swab test
ST: Serological test
JSS: Jeskin Sleep Scale
PSS: Perceived Stress Scale
SD: Standard deviation
CMV: Cytomegalovirus
HPV: Human papillomavirus
AST: Aspartate aminotransferase
ALT: Alanine transaminase
PTH: Parathyroid hormone
TSH: Thyroid-stimulating hormone reflex
AbTPO: Thyroperoxidase antibodies
ACE2: angiotensin converting enzyme 2
CRP: C-reactive protein
IL-6: Interleukin 6
SREBP-1: sterol regulatory-element binding protein-1
EU-GDPR: EU General Data Protection Regulation
