# Supplementary material for: The EPICOVID19-BS study: a web-based epidemiological survey in bariatric patients
Source: J Endocrinol Invest. 2024 Jun 10;48(1):173–89. doi: 10.1007/s40618-024-02407-1 (PMC11729083; doi:10.1007/s40618-024-02407-1)
Supplement: Supplementary file 1 — Supplementary file1 (DOCX 19 KB) [file 40618_2024_2407_MOESM1_ESM.docx]

**Supplementary material**

**Table 1. Distribution of symptoms by COVID-19 (n=745)**

|  | **COVID-19** | |  | ***p-value*** |
| --- | --- | --- | --- | --- |
|  | **No** | **Yes** | **Total** |  |
| Fever | 53 (8,9) | 79 (52,0) | 132 (17,7) | <0,001 |
| Cough | 44 (7,4) | 57 (37,5) | 101 (13,6) | <0,001 |
| Sore throat/Rhinorrhea | 88 (14,8) | 67 (44,1) | 155 (20,8) | <0,001 |
| Headache | 95 (16,0) | 79 (52,0) | 174 (23,4) | <0,001 |
| Myalgia | 81 (13,7) | 88 (57,9) | 169 (22,7) | <0,001 |
| Dysgeusia | 17 (2,9) | 74 (48,7) | 91 (12,2) | <0,001 |
| Anosmia | 16 (2,7) | 73 (48,0) | 89 (11,9) | <0,001 |
| Shortness of breath | 16 (2,7) | 39 (25,7) | 55 (7,4) | <0,001 |
| Chest pain | 13 (2,2) | 16 (10,5) | 29 (3,9) | <0,001 |
| Gastrointestinal | 46 (7,8) | 44 (28,9) | 90 (12,1) | <0,001 |
| Dermatological | 12 (2,0) | 5 (3,3) | 17 (2,3) | 0.351 |
| Loss of appetite | 7 (1,2) | 32 (21,1) | 39 (5,2) | <0,001 |
| Cardiological | 14 (2,4) | 14 (9,2) | 28 (3,8) | <0,001 |
| Neurological | 15 (2,5) | 12 (7,9) | 27 (3,6) | 0,002 |
| No symptom | 421 (71,0) | 28 (18,4) | 449 (60,3) | <0,001 |

**Table 2 – Positive Nasopharyngeal Swab, hospitalizations and drugs (n=126)**

|  |  |  |
| --- | --- | --- |
|  |  | **Total** |
| Type of NPS | Molecular | 97 (77,0) |
|  | Rapid | 27 (21,4) |
|  | Does not know | 2 (1,6) |
| Positive NPS performed because of |  |  |
| Symptoms |  | 83 (65,9) |
| Contact with COVID case |  | 57 (45,2) |
| Check at workplace |  | 11 (8,7) |
| Own choice |  | 3 (2,4) |
| Other reasons |  | 7 (5,6) |
| Within 2 weeks before positive NPS, attending |  |  |
| Schools |  | 14 (11,1) |
| Restaurants |  | 40 (31,7) |
| Disco |  | 9 (7,1) |
| Churches |  | 3 (2,4) |
| Hairdressers |  | 20 (15,9) |
| Cinemas |  | 1 (0,8) |
| Parties |  | 24 (19,0) |
| Shared workplace |  | 55 (43,7) |
| Went to the hospital after a positive NPS | No | 114 (90,5) |
|  | Yes, immediately discharged | 5 (4,0) |
|  | Yes, hospitalized, no ventilation | 6 (4,8) |
|  | Yes, hospitalized, NIV | 1 (0,8) |
| Length of hospitalization in days |  | 9,9 ± 6,6 |
| Heparin |  | 22 (17,5) |
| Corticosteroids |  | 34 (27,0) |
| Azithromycin |  | 19 (15,1) |
| Other antibiotics |  | 23 (18,3) |
| Antipyretic |  | 38 (30,2) |
| Remdesevir |  | 1 (0,8) |
| Kaletra |  | 0 (0,0) |
| Cloroquine |  | 1 (0,8) |
| Oxygen therapy |  | 5 (4,0) |
| Other/Does not know |  | 44 (34,9) |
| NPS control performed | No | 12 (9,5) |
|  | Yes, the most recent NPS was negative | 102 (81,0) |
|  | Yes, the most recent NPS was positive | 12 (9,5) |
| Time since first positive NPS to last positive NPS in days |  | 27,5 ± 30,8 |
| Time since first positive NPS to first negative NPS in days |  | 20,4 ± 31,2 |
| No NPS control performed | After less than one month since the first positive NPS | 5 (41,7) |
|  | After more than one month since the first positive NPS | 7 (58,3) |
